# Scientific Versus Experiential Evidence: Discourse Analysis of the Chronic Cerebrospinal Venous Insufficiency Debate in a Multiple Sclerosis Forum

**DOI:** 10.2196/jmir.4103

**Published:** 2015-07-01

**Authors:** Janka Koschack, Lara Weibezahl, Tim Friede, Wolfgang Himmel, Philip Makedonski, Jens Grabowski

**Affiliations:** ^1^ Department of General Practice University Medical Center Göttingen Göttingen Germany; ^2^ Department of Medical Statistics University Medical Center Göttingen Göttingen Germany; ^3^ Institute of Computer Science Georg-August- University Göttingen Göttingen Germany

**Keywords:** multiple sclerosis, venous insufficiency, Internet, social media, cognitive dissonance, qualitative research

## Abstract

**Background:**

The vascular hypothesis of multiple sclerosis (MS), called chronic cerebrospinal venous insufficiency (CCSVI), and its treatment (known as liberation therapy) was immediately rejected by experts but enthusiastically gripped by patients who shared their experiences with other patients worldwide by use of social media, such as patient online forums. Contradictions between scientific information and lay experiences may be a source of distress for MS patients, but we do not know how patients perceive and deal with these contradictions.

**Objective:**

We aimed to understand whether scientific and experiential knowledge were experienced as contradictory in MS patient online forums and, if so, how these contradictions were resolved and how patients tried to reconcile the CCSVI debate with their own illness history and experience.

**Methods:**

By using critical discourse analysis, we studied CCSVI-related posts in the patient online forum of the German MS Society in a chronological order from the first post mentioning CCSVI to the time point when saturation was reached. For that time period, a total of 117 CCSVI-related threads containing 1907 posts were identified. We analyzed the interaction and communication practices of and between individuals, looked for the relation between concrete subtopics to identify more abstract discourse strands, and tried to reveal discourse positions explaining how users took part in the CCSVI discussion.

**Results:**

There was an emotionally charged debate about CCSVI which could be generalized to 2 discourse strands: (1) the “downfall of the professional knowledge providers” and (2) the “rise of the nonprofessional treasure trove of experience.” The discourse strands indicated that the discussion moved away from the question whether scientific or experiential knowledge had more evidentiary value. Rather, the question whom to trust (ie, scientists, fellow sufferers, or no one at all) was of fundamental significance. Four discourse positions could be identified by arranging them into the dimensions “trust in evidence-based knowledge,” “trust in experience-based knowledge,” and “subjectivity” (ie, the emotional character of contributions manifested by the use of popular rhetoric that seemed to mask a deep personal involvement).

**Conclusions:**

By critical discourse analysis of the CCSVI discussion in a patient online forum, we reconstruct a lay discourse about the evidentiary value of knowledge. We detected evidence criteria in this lay discourse that are different from those in the expert discourse. But we should be cautious to interpret this dissociation as a sign of an intellectual incapability to understand scientific evidence or a naïve trust in experiential knowledge. Instead, it might be an indication of cognitive dissonance reduction to protect oneself against contradictory information.

## Introduction

Patients increasingly search the Internet for information on medical conditions, including clinical news and treatment options. Although this information is typically provided by medical experts or commercial sources, there has also been an increase in peer-to-peer health care [1]. Not only evidence from scientific sources, such as the latest results of clinical trials, diffuses into the lay community in this way. Patients also share the experiences they have with doctors, treatments, and the everyday living with a disease with other patients in online forums [2]. These shared patient experiences have formed a new database of experiential knowledge that is not only a source of information for patients and their relatives, but also has increasing relevance for scientific research [3].

The patients’ use of the Internet as a source of both scientific and experiential knowledge is a cause of serious concern when these different forms of knowledge do not peacefully coexist, but are contradictory. This was recently observed in the debate about the endovascular treatment of multiple sclerosis (MS), which is based on a new etiologic hypothesis called chronic cerebrospinal venous insufficiency (CCSVI) [4]. Although the scientific community was opposed to or ignored the CCSVI hypothesis, it was heatedly debated in online patient communities, particularly the resulting treatment (ie, modified venous angioplasty or stenting of jugular and azygous veins) [5]. Although scientists and clinicians strongly advised against this procedure before it was rigorously scientifically examined for efficacy and safety [6], patients all over the world found a way to obtain access to this procedure, often commercially referred to as the “liberation treatment” [7]. Patients’ experiences with the liberation treatment were soon published on the popular video-sharing website YouTube. Mazanderani and colleagues [8] showed in a content analysis of these YouTube videos that patients used the videos to prove the effectiveness of the treatment, for instance, by showing improved symptoms after partaking in the treatment.

The debate about the CCSVI hypothesis and the associated intervention took place in countless patient forums all over the world. However, particularly in Canada, the demand of the patient community and its advocates for further research was so strong that CCSVI and the liberation treatment became a research topic, not defined by the scientific community itself, but by the patient community [9]. The CCSVI hype has abated somewhat since the most recent studies found neither evidence for a high prevalence of CCSVI nor for a causal relationship to MS, and a wave of complications following venous stenting and angioplasty was reported instead [10].

The CCSVI story is now seen as a “waste of valuable time, money, and intellectual energy” [11], at least by large parts of the scientific community. We know that the Internet and Web 2.0 played an important role in mobilizing thousands of participants in the CCSVI debate [12]. What we do not know is whether patients felt a conflict between their own understanding of evidentiary value and the agenda of the scientific community during the CCSVI debate and, if so, how they reconciled conflicting opinions, interests, and objectives.

By analyzing the CCSVI discussion in the patient online forum of the German MS Society (Deutsche Multiple Sklerose Gesellschaft; DMSG), we aimed to reconstruct the underlying discourse that forms this discussion. One approach to uncover discourses was introduced by the Duisburg School of Critical Discourse Analysis [13]. It is based on Michel Foucault’s discourse theory that deals with questions such as what knowledge is, how the valid knowledge evolves, which function it has for the constitution of subjects, and the shaping of society. Knowledge means all kinds of meanings used by real persons to interpret and shape the surrounding reality.

## Methods

### Database General Description

The database for this analysis consisted of contributions posted in the online forum of the DMSG. The DMSG presents itself on its website as a nonprofit stakeholder of MS patients and their relatives. It is a registered charity with more than 900 community contact groups. Among other features, the DMSG provides 2 different types of freely accessible forums on its website: an expert forum with time-limited chats between experts and users about different issues (eg, cognitive deficits or pregnancy) and a second forum that is unstructured, not moderated, and open for anonymous registration. It is targeted at laypeople, mostly patients with MS. The forum consists of threads. A thread is a . These postings can contain hyperlinks and can cite any number of previous postings. Every user can open a new thread or contribute to an existing one.

#### Data Reduction

**Figure 1:**
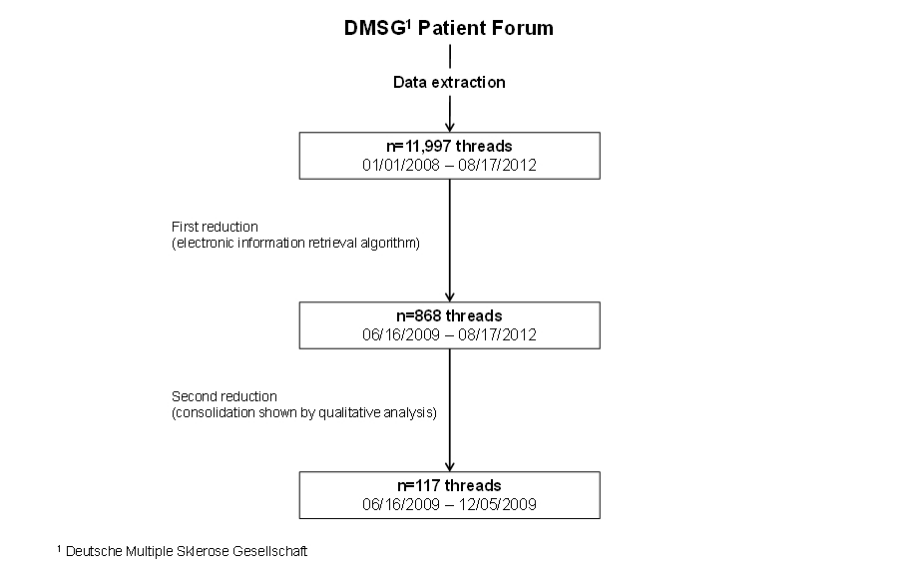
Flowchart of the data extraction and reduction process.

Initially, all contributions between January 1, 2008 (the starting point of the forum) and August 17, 2012 (the date of the extraction) were extracted. This initial database consisted of 139,912 postings and was reduced first to postings contributing to the CCSVI discussion. The information retrieval algorithm for identifying individual postings is described in detail for the quantitative analysis [14]. A total of 868 CCSVI-related threads containing more than 53,000 postings were identified. The threads varied by numbers of postings; a few contained only one posting and the longest had more than 2000 postings. The first CCSVI-related posting determined the beginning of our data analysis. To define the end of the chronological analysis, we followed the concept of saturation, a guiding principle in qualitative research. Our sample had to be large enough to assure that most or all the perceptions that might be important were uncovered, but at the same time if the sample was too large, the data would become redundant. Thus, we looked for a consolidation on levels such as contributors, statements, and argumentations. Six months after the first CCSVI-related posting, we could not identify further discourse fragments that provided new information or put the data already gathered into perspective. The flowchart in Figure 1 shows the steps of the data extraction and reduction.

### Analysis

Many critical discourse analyses deal with text that is produced under certain formal criteria and related to a specific topic (ie, newspaper articles about the increasing rate of childhood obesity). In this example, the newspaper articles are the textual elements to a particular topic and are called “discourse fragments.” These discourse fragments form the “discourse strand” (in this example, the debate about childhood obesity). In our analysis, we defined “CCSVI” as the discourse strand formed by the associated discourse fragments, (ie, the threads related to this topic). Jäger [13] suggests a sequential procedure with 2 main steps: (1) structural analysis of the discourse strand and (2) detailed analysis of typical discourse fragments, including context and surface of the thread, content of the thread, ideological statements in the associated postings, and use of collective symbolism in the associated postings.

#### Structural Analysis of the Discourse Strand

We read and reread the CCSVI-related threads of the patient forum in a chronological order. We identified significant users (ie, users who posted very often) or whose postings started lively discussions. These users also gave us a preliminary idea of discourse positions that would need to be highlighted and defined in the next step of the analysis. We gathered themes in the discourse that were discussed repeatedly and tried to determine their meaning and impact on the discourse. Three characteristics seemed especially promising for identifying discourse fragments (ie, threads) as typical for the discourse strand: (1) when they started a lively discussion in the forum, meaning that many different users with different opinions reacted to that posting; (2) when they triggered certain (announced or reported) actions in real-life, such as talking with one’s own doctor about CCSVI; and (3) when they activated a certain style or nature of the discussion (eg, polemic argumentation).

#### Detailed Analysis of Typical Discourse Fragments

##### Context and Surface of the Thread

We described the thread, including the number of contributing users, whether it was from a well-known user (ie, from a user who often posted and elicited many responses from other users), the number of postings, the course of the thread (eg, whether it meandered or was concise), and whether it mentioned subtopics that were discussed previously. We then described the context of the thread, meaning information about whether the thread was triggered by a real-life event. We also reported any characteristics of the thread that might be important for interpreting it.

##### Content of the Thread

We analyzed the thread chronologically to reveal its logical structure. Subtopics were scrutinized for their meaning (ie, which emotions and connotations were addressed) and how a subtopic was related with other subtopics.

##### Ideological Statements in the Associated Postings

We analyzed the arguments used to justify attitudes or emotions. Arguments were deconstructed into their superficial content (eg, “the subgroup of primary-progressive MS patients is neglected”) and we flagged the strategy that was used to prove the content (eg, by quoting an expert who stated it). We then determined certain underlying dimensions or features that help reconstruct the different discourse positions from which the forum users evaluated the CCSVI debate.

##### Use of Collective Symbolism in the Associated Postings

We determined cultural stereotypes, such as figures of speech and allegories, that seemed to be a common ground for the users in the forum, signified something specific, and were therefore able to popularize knowledge.

We followed Jäger’s suggestion of a cyclic and iterative working to reveal connections between different aspects of the analysis, to develop interpretations, pinpoint arguments for or against these interpretations, and to reconstruct certain discourse positions from which subjects participate in and evaluate the discourse.

Critical discourse analysis as an interpretative method does not need to present quotations as proof or examples as is commonly done in other qualitative approaches, such as in a content analysis. We only present quotations in one case to illustrate the use of collective symbolism. To demonstrate our methodical approach, Textbox 1 provides an example of the detailed analysis of a typical discourse fragment [15]. The thread was translated from German into English and shortened for demonstration purposes. Main contributors to the qualitative analysis were 3 of the authors (LW, WH, JK). Analysis was done in a team approach, so differences were resolved by in-depth discussion and consensus was sought during the process of analysis. Important intermediate results were discussed with the other 3 authors.

**Detailed analysis of a thread [16] as a typical discourse fragment (thread translated and shortened to the first 2 postings).**

Post 1: “Dr XXX’s answer to questions concerning venous MS makes the distance abundantly clear that certified doctors have to existential interests of MS patients! The status quo is just right and comfortable. Please do not disturb! I don’t know whether the story about the veins is correct, but the lack of willingness to actually apply oneself to this subject clearly shows that there’s nothing to be expected from the certified angle, not even disproving seems to be necessary. I sincerely thank you, Dr XXX, for this honest confession (a bitter truth for us). I’ve never heard it admitted this openly.” (User 1, 11:40 am September 18, 2009)

Post 2: “These gods in white don’t know a thing, they just read the press releases of the pharma mafia about just how potent these dubious BT are.” (User 2, 00:19 pm September 18, 2009)

Thread title: “The overstrained/threadbare expert” [German: “Der überstrapazierte Experte”]

Time period: First posting 11:40 am on September 18, 2009; last posting 2:25 pm on September 19, 2009

Participants: 18 users; several well-known users (regular contributors) of the forum (eg, User 1, User 2 [usernames replaced with placeholders])

Thread characteristics: 28 postings; a short, but very dense thread dealing with the following subtopics: “who is the expert,” “Big Pharma,” “what constitutes trustworthiness,” and “DMSG no patients advocate”

Context and content: Thread is about an event from outside the patient forum, but within the DMSG website: Three days before (8:02 pm September 15, 2009), the editorial office deleted some postings addressed to an expert together with the expert’s answers. The office explained this removal as follows: The postings were not dealing with the announced topic of the expert forum (ie, “Different courses—different therapies”). Instead, the users consulted the expert about CCSVI.

Description (of the first 2 postings): User 1 (well-known; patient with primary-progressive MS [PPMS]; already suffering from some disabilities) starts with a quotation of Dr. XXX [not printed here]. He interprets Dr. XXX’s statement as exemplary for the dissociation between the needs of the patient community and the attitudes of the expert community. He uses the attribute “certified” for the medical profession—although a doctor is qualified per se, there is no additional certification needed. User 1 stated his frustration about the expert’s lack of interest to discuss CCSVI and he interpreted this lack of interest less as an opinion about CCSVI, but more as the typical arrogant stance experts have toward patients. Thirty minutes later, User 2 responded to User 1′s posting (User 2 is also well-known; MS patient with PPMS; already suffering from some disabilities). User 2 uses the [German] phrase “gods in white,” which expresses the widely shared opinion that doctors are almost almighty, but they do not share the afflictions of common people at all. “White” in this phrase refers to the color of the commonly worn doctor’s coat. His negative judgment is fueled by the postpositive statement that doctors are naive because they uncritically believe the pharmaceutical industry’s advertising of “BT” (BT is the abbreviation of beta-interferon, the active ingredient in the currently most commonly prescribed medication for relapse-remitting MS in Germany).

Comments (about the first 2 postings): The thread title uses a German phrase that can be interpreted in 2 ways: as “overstrained” (meaning that the expert is unable to answer adequately for various reasons) vs “threadbare” (ie, the expert is not an expert at all, his declared status as an expert is a farce).

The thread title already shows the ironically contested expert status of someone who is in fact an expert; thus, expertise as a sign of quality for health care is doubted (subtopic “who is the expert”).

User 1 must assume that he is perceived as trustworthy by the other users because the posts are already deleted, it is not possible to determine whether this is true—trustworthiness as a matter of personal involvement and being recognizable as an individual (subtopic “what constitutes trustworthiness”).

By doubting Dr. XXX’s expert status, the expert status of the DMSG is also disputed because Dr. XXX is a member of the DMSG advisory board (subtopic “DMSG no patients advocate”)

User 2 shows by using the abbreviation “BT,” which only insiders know about, that he considers himself some kind of expert with special knowledge (subtopic “who is the expert”).

Doctors are not seen predominantly as selfish betrayers, but as being caught by their own arrogance not to see that they are as framed as the patients by the pharmaceutical industry (subtopics “Big Pharma” and “who is the expert”).

Collective symbolism (in the first 2 postings): “God in white”: a stylistic device to ironically qualify doctors as arrogantly believing being capable of everything (ie, like a god).

### Ethical Consideration

There are no rules for the ethical challenge that is inherent to using health discussion board postings as research data [16]. Because we were unable to obtain informed consent from the forum visitors to use the data they produced by posting to the forum, we officially informed the executive board of the DMSG about the study and they gave us their consent. Additionally, we asked the Ethics Committee of the University Medical Center Göttingen for approval. The committee decided that an approval was not necessary (11/5/13An). In the rare cases of using quotations, we used pseudonyms instead of real usernames or replaced the real names with XXX, respectively.

## Results

The first posting related to CCSVI was published on June 16, 2009, and we completed our analysis with data from the end of 2009. At that time, the CCSVI discourse consisted of 117 threads (1907 postings) (see Figure 1). We identified 2 main discourse strands. Some collective symbols attracted our attention during the detailed analysis. Finally, we disclosed certain dimensions of argumentation and reconciliation that apparently were useful for interpreting discourse positions within the CCSVI discourse.

### Discourse Strands

We identified certain subtopics in the threads, such as “who is the expert,” “Big Pharma,” and “what constitutes trustworthiness” (see Textbox 1). By determining the relationship between these subtopics, we reconstructed 2 discourse strands: (1) the “downfall of the professional knowledge providers” and (2) the “rise of the nonprofessional treasure trove of experience.”

#### The Downfall of the Professional Knowledge Providers

The first posting mentioning CCSVI was answered 5 hours later with a link to the original CCSVI study. Like scientists, some of the users began to discuss the CCSVI hypothesis and Zamboni’s study [4] against the background of evidence-based medicine, using terms such as “placebo,” “number of cases,” and “blinded.” However, this parascientific discourse was not continued; the lack of any further evidence-based information may be the reason. Although users clearly understood that it was necessary to have more evidence, some of them argued that the progressive course of their disease did not to allow them to wait until the scientific community produced better knowledge.

Because actual evidence-based knowledge was lacking at that time, the archive of scientific knowledge was examined and an older publication from 1986 was introduced [17]. The long time between the first idea of MS of a vascular disease in 1986 and the new article more than 30 years later caused some users to ask why this theory had not been proven in the past—their answer was that opportunistic interests had blocked and hindered further scientific investigation. Reasonable doubts regarding the scientific validity of the hypothesis were not discussed.

This feeling of distrust of the scientific community or, more precisely, of the well-established neurological scientific community tainted the ongoing discussion and the following events were mainly interpreted as confirmation of this feeling. In August 2009, 2 months after the first CCSVI-related posting in the forum, a conference was held by Zamboni himself in Bologna, Italy, raising the users’ hope that CCSVI now would become an important topic in the scientific community. This conference was largely ignored by the scientific community, which was interpreted as more proof that mainstream research in MS was not a stakeholder of patient interests. The forum users did not discuss that the conference lacked the common attributes of a scientific convention: only researchers who collaborated with Zamboni took part (ie, no scientists with a negative opinion about CCSVI). Users instead interpreted the situation as evidence for the ignorant mainstream knowledge providers.

#### The Rise of the Nonprofessional Treasure Trove of Experience

From the beginning of the CCSVI debate, forum users tried to validate the CCSVI hypothesis against their knowledge about MS in general (eg, by citing epidemiological facts such as the unequal sex ratio in MS) and their own illness experiences, such as symptom improvement by certain yoga techniques that are claimed to alter blood flow. In parallel to this embedding of CCSVI into the existing knowledge, users began to construct new experience-based knowledge about CCSVI. Two months after the first CCSVI-related posting, they began to publish first- or secondhand results of diagnostic and therapeutic procedures in the forum. In addition to this, they created hyperlinks to YouTube videos from MS patients from other parts of the world, which showed the CCSVI treatment and its positive outcome and also boosted the amount of experience-based knowledge about CCSVI when the evidence-based knowledge of the scientific community remained absolutely static without any new empirical results.

### Collective Symbols

Many contributions to the CCSVI debate were colored by emotions, as could be observed from the frequent use of collective symbols, such as the German figure of speech “Halbgott in Weiß” (“demigod in white”) used for degrading medical experts (see Textbox 1). Another example was the repeated use of “Bahnhof” (“railroad station”), a metaphor with connotations of “getting lost” or “being left behind.”

We analyzed one figure of speech to reveal the underlying images from which the users constructed the picture of CCSVI and MS. The German expression “eine neue Sau durchs Dorf treiben” literally translates as “to chase a new sow through the village” and means to make a big fuss about something new, with a clearly negative connotation, and is often associated with the feeling that it distracts the audience’s attention from the topic that really matters. In the context of CCSVI, the expression was always used to describe exactly this; the course of the CCSVI debate was sensed as familiar and repetitive, such as many other etiologic or therapeutic breakthroughs that were unable to keep the promise to heal MS. For some figures of speech, English-German matches exist. For example, “the early bird catches the worm” can be literally translated into German “Der frühe Vogel fängt den Wurm.” In our case, it would be impossible to translate the figure of speech literally. Therefore, we give the original German quotation and its English denotation:

...In ein paar Monaten wird die Sau, die jetzt noch durchs MS-Dorf getrieben wird, tot zusammenbrechen, wie alle Wunderkur-Säue zuvor. Als alte MS-Hasen haben wir schon viele Säue verrecken sehen...[...few months from now, this final cure will all be revealed to be much ado about nothing, as were all the other final wonder-cures before. We’ve tried so many final cures before, we who’ve had MS for such a long time...]

User 3, posted 1:08 pm October 17, 2009

Stammzellen waren doch gestern. Heute sind’s Krampfadern im Oberstübchen. Und morgen ne neue Sau, die durchs Dorf getrieben wird. Man darf also gespannt sein...[Stem cells are yesterday’s news, aren’t they. Today it’s varicose veins in the belfry. And tomorrow it will be a new final cure. We’re all absolutely holding our breath...]

User 4, posted 10:46 pm August 16, 2009

...Die “Krampfadern-im-Gehirn-Hypothese” kommt alle paar Jahre wieder, wenn wieder ein neues Publikum herangewachsen ist. Mal sehen, wie lang es diesmal dauert, bis die Sau sich durchs Dorf müde gerannt hat...[...The hypothesis of varicose veins in the belfry is repeated every few years when there’s been a generation change in the auditorium. It’ll be thrilling to watch how long it takes this time round until the bluff on the new final cure is called...]

User 5, posted 10:41 am July 29, 2009

The figure of speech portrays a certain emotional tableau of being disenchanted, hopeless, and being tired from past disappointments:

...Herzchen, die venöse Stau-Sau, die vielerorts getrieben wurde, ist hier schon durch. Wir haben sie gesehen, wahrgenommen, beklatscht, gewogen, für zu leicht befunden und in den nächsten Ort gejagt. Wenn Du Dich beeilst, dann kann es sein, dass Du sie noch einholst...Viel Glück. [...honey, the varicose-belfry apparition that’s been reported from many locations has been through here already. We’ve seen it, noticed it, applauded it, finally weighed it and found it wanting. Then we chased it to the next town. If you make haste, you might still be catching up with it...Good luck.]

User 6, posted 7:34 pm September 3, 2009

Additionally, this enables the poster to distinguish him- or herself from those who do not notice that they are being messed with:

...Bei aller Verzweiflung, die man als MS-Kranker so hat: man muss nicht jeder Sau hinterherrennen, die grade mal wieder durchs Dorf getrieben wird...[...Even considering the heights of desperation that one experiences as an MS patient: you don’t need to follow every self-proclaimed savior...]

User 7, posted 12:37 pm October 17, 2009

### Characterization of Discourse Positions

We detected 4 different positions from which users participated in the CCSVI discussion and evaluated the associated incidents: “hostile,” “frustrated,” “wait and see,” and “enthusiastic.” These positions were not exclusive, meaning that there was a possibility to switch from one position to another: The hostile and the frustrated position formed a counterpart to the wait-and-see and enthusiastic positions; switching within these 2 groups, but not between them, seemed to be possible.

The positions differed in their orientation toward or against evidence-based and experience-based knowledge as reflected by the discourse strands described previously. The need for evidence-based information to assess CCSVI in its diagnostic and therapeutic value seemed at first glance to be accepted by most users. However, the argument that only prospective studies conducted by high-class research institutes would be able to produce reliable evidence-based information was disputed. This objection was not the result of a negative attitude toward research in general. Instead, the time that research needed to produce evidence-based knowledge was considered a price not every patient could afford to pay. Thus, the trust in evidence-based knowledge and the time pressure perceived simultaneously caused some inconsistency that was associated with negative feelings toward scientific research and scientists.

During lively debates of the significance of individual experiences or the trustworthiness of scientific information, the discussion became often highly emotional. These feelings colored the arguments or were directly verbalized. At some points in the discussion, the emotional coloring developed into a subjectivity, which often manifested itself in insults against others. A conspiracy theory seemed to exist on both sides: The pro-CCSVI side contested “Big Pharma” to the point of felonies like murder. However, the anti-CCSVI side also doubted the motives of CCSVI-promoting doctors and scientists. Economic interests were the main argument of both sides. Emotionality or subjectivity seemed to mask a deep personal involvement.

Considering the collective symbols, the figure of speech “to chase a new sow through the village” emblematically portrayed the discourse positions that were hostile to the enthusiastic position: the posters were considered disenchanted but wise in contrast to those who were enthusiastic but foolish. Table 1 shows the 4 discourse positions arranged according to the dimensions “trust in evidence-based knowledge,” “trust in experience-based knowledge,” and “subjectivity.”

**Table 1:** Characterization of discourse positions during the chronic cerebrospinal venous insufficiency debate in the Deutsche Multiple Sklerose Gesellschaft (German Multiple Sclerosis Society) patient forum.

Dimension	Position			
	Hostile	Frustrated	Wait & see	Enthusiastic
Trust in evidence-based knowledge	Low	Low	Moderate	Moderate
Trust in experience-based knowledge	Low	Moderate	Moderate	High
Subjectivity	High	Low	Low	High

## Discussion

In the discourse analysis of the CCSVI discussion in a German MS patient forum, we tried to reveal how patients reconcile the controversial scientific CCSVI debate with their own illness experience. The users heatedly debated whether scientific results or experiences of other patients had value for their own opinion-making about CCSVI. By determining the relation between relevant subtopics, we could generalize 2 discourse strands: (1) the “downfall of the professional knowledge providers” and (2) the “rise of the nonprofessional treasure trove of experience.” The discourse strands indicated that the discussion moved away from the question whether scientific or experiential knowledge had more evidentiary value. Rather, the question whom to trust (ie, scientists, fellow sufferers, or no one at all) was of fundamental significance. Four discourse positions could be identified by arranging them to the dimensions of “trust in evidence-based knowledge,” “trust in experience-based knowledge,” and “subjectivity.” The emotional character of contributions was manifested by the use of popular rhetoric that seemed to mask a deep personal involvement.

At first glance, the lay discourse about the evidentiary value of experiential knowledge and the strong personal involvement may be interpreted as a misunderstanding of scientific methods, an intellectual incapacity to understand research practice, or even as irrationality. But this would be misjudging the discourses in patient online forums. Although most users were aware of the different positions in the scientific debate, at the same time they felt trapped in a conflictual relation between scientific and experiential knowledge, often associated with negative feelings of being cheated, left alone, and without control of their illness and their life. For that reason, the lay discourse in patient online forums should be interpreted as the result of the psychological efforts patients make to solve the conflictual tension arising from contradictory information.

### The Tension Between Scientific Evidence and Personal Experience

The amount of and access to health information on the Internet is growing exponentially and numerous studies have investigated how well laypeople can assess and evaluate the quality of this information. These studies showed that laypeople only infrequently check the source and date of health information [18], can be misguided by search machines [19], and often misunderstand clinical concepts and aims of clinical research studies [20]. However, in our analysis of the CCSVI debate, the users of the MS forum sophisticatedly discussed the advantages and disadvantages of scientific research and the quality criteria of clinical studies, and they also searched for latest results of clinical research in scientific databases. At this point, the ideal of the “expert patient”—a term appearing for the first time in a report presented to the UK Parliament in 1999 as a “healthy citizen” initiative to help deal with chronic illness [21]—seems to come true. In addition to pursuing scientific knowledge, the users have gained experiential knowledge by (1) making various personal experiences by themselves as patients suffering from MS and (2) listening to the experiences of other patients. We could reveal that the users showed both scientific and experiential knowledge in the CCSVI discussion at different levels of elaboration and deliberation.

However, we witness a tension that emerges between these 2 different forms of knowledge. This tension can also be observed when health care professionals feel confronted with scientific evidence that disputed the medical practices they had been used to for years [22,23]. Interestingly, since the beginning when evidence-based medicine (EBM) was implemented as the new paradigm of health care, skeptical voices have discussed the problems resulting from its one-dimensional interpretation of evidence as scientific evidence [24].

Compared to this epistemological debate about EBM, our analysis of the CCSVI discussion in a patient online forum showed similar reactions using a somewhat different language. The result was the same (ie, a conflict between scientific and experiential knowledge). Although several users called for more time to give scientific proof a chance, others were immediately enthusiastic about the promises of the liberation treatment. The users seemed to sense an obligation to choose between the 2 forms of knowledge. Both parties claimed the higher evidentiary value of their knowledge.

The competition between these 2 forms of knowledge has a long tradition, with formally acquired knowledge―typified by objective science―being valued and naturalistic knowledge―typified by subjective experience―being devalued. To paraphrase Peter Storkerson, formal methods of knowledge acquisition are equated with rigor and validity and, consequently, knowledge derived from the application of such methods is valued per se as the gold standard of evidence [25]. In contrast, experiential knowledge is associated with unconscious, nonconscious, or implicit thinking that does not involve explicit, expressible, analyzable theoretical systems of knowledge. However, the users, or at least some of them, ascribed evidentiary value to experiential knowledge and to scientific knowledge. This could be interpreted as a kind of justification to choose the party of experiential knowledge. This evidence could be called “experiential evidence.”

The conflictual tension between scientific and experiential evidence can be interpreted as “cognitive dissonance” [26], a discrepancy between action (ie, a forced choice between 2 alternatives) and attitude (ie, judging the 2 alternatives as being of the same value). Experimental social psychology has shown that people adjust their attitudes to support their decision by increasing their preference for the selected option, decreasing their preference for the rejected option, or both. This adjustment or rationalization is motivated by the urge to reduce the cognitive dissonance [27]. The theory of cognitive dissonance has been proven useful also in the context of health care research, for example, to examine patient behavior and emotional state after decisions concerning diagnostic or therapeutic procedures [27-29]. During the CCSVI debate, users might feel forced to choose between 2 alternatives of the same value on different levels: belief in scientific versus experiential evidence and also intellectual skepticism versus desperate hope.

### A Matter of Trustworthiness

While discussing facts about and experiences with CCSVI in the forum, users were concerned about the quality of the information. They doubted that the DMSG was really neutral and patient-oriented; its impersonal stakeholders were assumed to be opportunistic and loyal to “Big Pharma.” This reservation was also expressed toward scientists. They were perceived and portrayed as controlled by the pharmaceutical industry and driven by economic and career-related motives. In parallel to the devaluation of the DMSG and the MS research community, some other players were valorized by crediting them with trustworthiness. An example is Paolo Zamboni, the leader of the CCSVI movement. His trustworthiness was confirmed for the forum users because his wife has MS—this message was frequently communicated in the forum. This personal involvement resulted in a presumedly altruistic motivation.

This process of valorizing and devaluing different sources of knowledge is again in accordance with results of social psychology and the theory of cognitive dissonance. One way to reduce cognitive dissonance is to challenge the source of the conflicting information (ie, to challenge its trustworthiness). Valorizing Zamboni by crediting him with trustworthiness was enhanced by the positive media presentation of Zamboni as the savior of his own wife and the liberation treatment like a miracle cure [30].

### Disenchanted but Wise Versus Enthusiastic but Foolish

The different argumentation strategies and attitudes were revealed as the basis for the construction of 4 discourse positions in the CCSVI forum debate: the positions “hostile” and “frustrated” were contrasted with “wait and see” and “enthusiastic.” These positions appear at first glance to be simple orientations or prejudices to uncritically adopt or reject new ideas. However, a more detailed examination showed that these argumentation strategies and attitudes were means to overcome the conflict between existing scientific results and experiential knowledge (ie, to reduce the cognitive dissonance). To reduce the cognitive dissonance in the CCSVI debate seemed to reflect a core experience in the course of the patients’ illness experience: either you choose to distrust every new therapy and hypothesis to avoid being disappointed yet again or you choose to trust every new therapy and hypothesis in order not to miss the opportunity to be healed. The cognitive dissonance was perfectly symbolized by the often-used figure of speech “eine Sau durch das Dorf treiben” (to chase a sow through the village) that describes the situation of MS patients as either disenchanted but wise or enthusiastic but foolish.

There seems to be a relation between the 2 discourse positions with high subjectivity (ie “hostile” and “enthusiastic”) and the level of personal involvement. We did not have any valid personal information about the contributors of the CCSVI discussion in the DMSG patient online forum (eg, age, sex, MS type). Statements about concrete relations between personal experiences as MS patient and the content and form of the contribution to the discussion would be highly speculative. However, reading between the lines (ie, when users casually talked about experiences they had as MS patients with the health care system and biomedical research) indicated different illness biographies. We sensed these illness biographies were dichotomized: biographies with a more benign course of the disease and positive experiences with academic medicine or biographies with a more devastating course and rather disappointing experiences with medicine and biomedical research. It might be speculated that the latter leads to a deeper personal involvement.

### Strengths and Limitations

There are many articles discussing the developments in the context of the CCSVI debate, mainly comments or editorials [7,9]. However, articles delivering empirical results are rare. In addition to our own exploratory analysis [13], to our knowledge there is only one study that empirically investigated the CCSVI debate in the patient community with methods of the social sciences. The authors showed that patient experiences published as YouTube videos may replace evidence-based scientific information or create a hybrid of personal experience plus medical knowledge [8]. Although this analysis classified videos by their thematic content, our discourse analysis adds another piece of the puzzle of better understanding how patients try to reconcile the discrepancies between different forms of evidence.

Our study has some limitations. When using data that naturally emerged versus data produced for study purposes, such as survey data, the contributors are unlikely to be a representative sample of the population being analyzed. It is very likely that our sample of German MS patients consisted of more patients with a primary-progressive MS (PPMS) course than is true for the German MS population as a whole. Patients with PPMS are more often therapy refractory and have a fatal course with a higher grade of disability in a shorter period of time than patients with a relapsing-remitting MS [31]. The relation between a higher grade of disability or a worse course of disease and a more active commitment in patient forums and other social media has been described in the literature [32]. Patients with PPMS may more often have reservations against the health care system, biomedical science, and pharmaceutical industry, so that the CCSVI debate in our forum might be biased to a more negative course. Second, the analysis was based on only one online forum for German-speaking MS patients. Differences in culture, health care systems, and available treatments may influence the course of the CCSVI debate.

It would have been interesting to analyze in which way the discussion about CCSVI developed in the forum when the users had to face scientific evidence against the hypothesis and—perhaps more important—the liberation treatment. However, our main interest was to elucidate what happens in the lay discourse in ambivalent scientific situations. Ambivalence could arise when there are conflicting scientific results about one issue (eg, such as the case of breast cancer) [33]. Another example is the situation when a new hypothesis about a disease and its treatment spreads from the scientific community into the patient and lay community at a point of time when large clinical studies providing scientific evidence are still lacking. This was the case of CCSVI in the first 18 months after the initial Zamboni publication. Therefore, we decided to focus our analysis on this period of time.

### Conclusions

We reconstructed a lay discourse of the evidentiary value of knowledge by critical discourse analysis of the CCSVI discussion in a patient forum and explained the development of the discourse with the theory of cognitive dissonance. This explanation puts the “expert patient” as proclaimed by health politics and research in question if “being an expert” means to be able to deliberate in cold blood the advantages and disadvantages of scientific evidence versus personal experience or to objectively evaluate conflicting results of scientific research. “Being a patient” means to be personally and emotionally affected, always “at risk of clutching at any straw.” A healthy person without a family history of cancer balancing the advantages and disadvantages of taking part in the screening program is in a completely different situation than someone who is already handicapped by an illness and must fear losing control over his or her life. The need for cognitive dissonance reduction in this situation is so complex and urgent that it would be an unacceptable simplification to interpret the contributions of the forum users as a sign of irrationality and intellectual incapacity of understanding scientific evidence or to blame them for trusting evidence that is based on experience.

Another unacceptable simplification might be the demand for science and scientists to regain lost credibility. The devaluation of science and scientists is probably not the result of the medial presentation of frauds and misconduct or the inability of scientists to explain their methods, results, and conclusions to the public. It is perhaps the consequence of patients’ drive to reduce the cognitive dissonance that results from the conflictual tension between scientific and experiential knowledge. However, the role of the media in the CCSVI debate, particularly in Canada, was a good example of how the press presents new and sometimes absurd scientific ideas as “breakthrough” and “new hope” [34]. It is very likely that the media presentation has aggravated the uncomfortable feeling of cognitive dissonance in MS patients and will aggravate the dissonance between hope and skepticism for many other patients in the future.

## Abbreviations

CCSVI: chronic cerebrospinal venous insufficiency
DSMG: Deutsche Multiple Sklerose Gesellschaft (German MS Society)
EBM: evidence-based medicine
MS: multiple sclerosis
PPMS: primary-progressive multiple sclerosis
